# The role of HIV infection in the etiology and epidemiology of diarrheal disease among children aged 0–59 months in Manhiça District, Rural Mozambique

**DOI:** 10.1016/j.ijid.2018.05.012

**Published:** 2018-08

**Authors:** Sozinho Acácio, Tacilta Nhampossa, Llorenç Quintó, Delfino Vubil, Charfudin Sacoor, Karen Kotloff, Tamer Farag, Nasrin Dilruba, Eusebio Macete, Myron M. Levine, Pedro Alonso, Inácio Mandomando, Quique Bassat

**Affiliations:** a Centro de Investigação em Saúde de Manhiça (CISM), Maputo, Mozambique; b Instituto Nacional de Saúde, Ministério de Saúde, Maputo, Mozambique; c ISGlobal, Hospital Clínic – Universitat de Barcelona, Barcelona, Spain; d Center for Vaccine Development (CVD), University of Maryland School of Medicine, Baltimore, MD, USA; e ICREA, Pg. Lluís Companys 23, 08010 Barcelona, Spain; f Pediatric Infectious Diseases Unit, Pediatrics Department, Hospital Sant Joan de Déu (University of Barcelona), Barcelona, Spain

**Keywords:** HIV, Moderate-to-severe diarrhea, Less-severe diarrhea, Mortality, Etiology, Children

## Abstract

**Background:**

Diarrhea is an important health problem among HIV-infected patients. This study evaluated the role of HIV in the epidemiology, etiology, and severity of diarrheal disease among children.

**Methods:**

The Global Enteric Multicenter Study enrolled children with moderate-to-severe diarrhea (MSD) and less-severe diarrhea (LSD) between December 2007 and November 2012. One to three controls for MSD cases and one per LSD case were enrolled and matched by age, sex, and neighborhood. All children were tested for HIV. Clinical data, anthropometric data, and stool samples were collected. Follow-up was performed at 60 days.

**Results:**

Two hundred and fourteen MSD cases and 418 controls, together with 349 LSD cases and 214 controls were tested. HIV prevalence was 25% among MSD cases (4% for matched controls) and 6% among LSD cases (6% among matched controls). HIV-infected children were more likely to have MSD (odds ratio 5.6, *p <* 0.0001).

Mortality rates were higher among HIV-infected children than among the uninfected (34 vs. 5 per 1000 child-weeks at risk; *p* = 0.0039). Cryptosporidium, Giardia, and enteroaggregative *Escherichia coli* (aatA only) were more prevalent among HIV-infected MSD cases than among uninfected ones.

**Conclusion:**

HIV is an important risk factor for MSD. The high mortality rate implies that children with MSD should be screened for HIV and managed accordingly.

## Highlights

• HIV prevalence was higher among cases with moderate-to-severe diarrhea (MSD) than controls.
• Mortality was higher among HIV-infected children with diarrhea than HIV-uninfected ones.
• HIV-infected children were more likely to have MSD.
• Cryptosporidium was the most common pathogen in HIV-infected children with MSD.
• *Escherichia coli* producing heat-stable toxin (enterotoxigenic *Escherichia coli*, any sequence type) was the most common pathogen in HIV-infected children with less severe diarrhea.

## Introduction

HIV is a significant public health problem worldwide. Globally, it is estimated that 2.1 million children under 15 years of age are infected by HIV, with about 160 000 new infections and 120 000 related deaths annually (World Health Organization, 2017a), the majority of which occur in Sub-Saharan Africa. Most of these children acquire the infection by vertical transmission from their HIV-infected mothers during pregnancy, delivery, or through breastfeeding (World Health Organization, 2017b). HIV-infected children become progressively immunosuppressed, a situation that increases their risk of acquiring common and life-threatening childhood infections (Humphreys et al., 2010).

The HIV pandemic has hit Mozambique particularly hard. This is currently one of the most affected countries in the world, with a nationally reported HIV prevalence in adults of 13% (Instituto Nacional de Saúde (INS) and Instituto Nacional de Estatística (INE), 2017), although community cross-sectional studies conducted in recent years in the district of Manhiça have repeatedly estimated the HIV prevalence in this specific district to be as high as 40% (Gonzalez et al., 2015, Gonzalez et al., 2012). In this setting, vertical transmission rates in the first month of life for children born to HIV-infected mothers have been estimated at around 9% (Moraleda et al., 2014).

Mozambique has implemented a series of diagnostic and preventive services to reduce the disproportionate burden imposed by HIV. Pregnant women attending antenatal clinics are routinely screened for HIV according to the national guidelines, and antiretroviral treatment (ART) is offered free of charge regardless of CD4 count, whenever the test is positive. Appropriate prophylaxis against vertical transmission or post-delivery ART treatment of the newborn is instituted for those children born to HIV-infected mothers, while awaiting definitive molecular confirmation of the infection (República de Moçambique et al., 2016). However, the prevention of mother-to-child transmission (PMTCT) remains suboptimal, and many incident infections still occur, leading to a relatively high HIV prevalence among children under the age of 5 years.

Similarly, diarrheal diseases still represent a major cause of morbidity and mortality in childhood, in spite of decreasing trends in recent years. Diarrheal diseases are estimated to account for around 578 000 annual child deaths, i.e. approximately 9% of all under-five deaths globally (Liu et al., 2015). The geographical distribution of these diarrhea deaths remains disproportionately shifted to resource-constrained settings, with Sub-Saharan Africa and Southeast Asia contributing to approximately 78% of deaths due to diarrhea worldwide (Humphreys et al., 2010, Boschi-Pinto et al., 2008). In these settings, challenging socio-economic and hygiene conditions, fragile health systems, and limitations in the deployment of already proven or existing preventive strategies such as oral rehydration solution (ORS) or enteric vaccines, all contribute to maintain the significant public health impact imposed by diarrhea (Liu et al., 2015, Bern et al., 1992, Kosek et al., 2003). Furthermore, the HIV/AIDS pandemic has significantly worsened the profile of diarrheal disease in these regions (Thea et al., 1993, van Eijk et al., 2010).

The etiological agents causing diarrhea vary greatly depending on the region of the world, but a recent multicenter study carefully characterized those of major relevance using a case–control approach enrolling not only moderate-to-severe diarrhea (MSD) or less-severe diarrhea (LSD) cases, but also matched healthy community controls (Kotloff et al., 2013, Liu et al., 2016). Diarrhea cases among HIV-infected children have been reported to be more severe, longer-lasting, cause longer hospital admissions, and be related to more co-morbidities and higher case-fatality rates (Groome and Madhi, 2012a, Chhagan and Kauchali, 2006, Keusch et al., 1992, Johnson et al., 2000). Related data regarding the specific etiology of diarrhea in HIV-infected children are still scarce for most of Sub-Saharan Africa. Previous mortality studies among young children with diarrhea in various settings in Africa have identified immunosuppression by HIV infection as an important factor for diarrhea and deaths (van Eijk et al., 2010).

In Manhiça District, in southern Mozambique, where HIV prevalence remains exceedingly high (Gonzalez et al., 2015, Gonzalez et al., 2012), diarrhea is the third leading cause of hospital admission among children 0–14 years and the fourth leading cause of death among children 12–59 months of age (Mandomando et al., 2007, Sacarlal et al., 2009, Nhampossa et al., 2015).

The aim of this study was to evaluate the role of HIV infection in modifying the frequency and etiology of MSD, and to determine its impact on mortality among children less than 5 years of age with diarrhea in Manhiça District, rural Mozambique.

## Materials and methods

### Study area and population

This study was conducted by the Centro de Investigação em Saúde de Manhiça (CISM) in Manhiça District, a rural area in southern Mozambique, under the framework of The Global Enteric Multicenter Study (GEMS) – “Diarrheal Disease in Infants and Young Children in Developing Countries” – coordinated by the Center of Vaccine Development (CVD) at the University of Maryland, Baltimore, Maryland, USA.

Manhiça District has an estimated population of 183 000 inhabitants. During the study period, more than half of the inhabitants were living in an area under continuous demographic surveillance as part of CISM Demographic Surveillance System (DSS). The area has been described in detail elsewhere (Sacoor et al., 2013). The CISM DSS is linked to a morbidity surveillance system at Manhiça District Hospital, the referral health facility for Manhiça District, and is also ongoing at five additional smaller health posts within the district (Guinovart et al., 2008, Bassat et al., 2011).

Manhiça District Hospital is a 110-bed referral hospital and has a 16-bed specific malnutrition ward. It admits an average of 3500 children annually. Standardized forms are routinely completed for all outpatients and inpatients, and include demographic, clinical, and laboratory data. Weight is measured for all outpatients, but height is only measured for admitted patients. Malaria is proactively screened in all patients with a fever or a referred history of fever in the preceding 24 h. Upon admission, a single blood culture is routinely performed for all children under the age of 2 years and for older children with a documented temperature ≥39 °C with severe malnutrition or other signs of severe disease, according to clinical judgment (Sigaúque et al., 2009).

### Data collection and clinical management

The GEMS case–control study was conducted between December 2007 and November 2012, and has been described extensively elsewhere (Kotloff et al., 2013). In brief, the study targeted three age strata: infants aged 0–11 months, toddlers aged 12–23 months, and children aged 24–59 months. All children in these age strata from the DSS population who sought care at the health centers within the DSS area were screened for diarrhea, defined as ≥3 loose stools within the preceding 24 h. Study clinicians assessed each child with diarrhea for eligibility. To be included, the episode had to be new (onset after ≥7 diarrhea-free days), acute (onset within the previous 7 days), and fulfill at least one of the following criteria for MSD: sunken eyes, loss of skin turgor (abdominal skin pinch with slow (≤2 s) or very slow (>2 s) recoil), intravenous hydration administered or prescribed, dysentery (visible blood in loose stools), or admission to hospital with diarrhea or dysentery. Additionally, a group of LSD cases, not fulfilling any of the aforementioned criteria for MSD, was also recruited (Kotloff et al., 2013, Kotloff et al., 2012). For each MSD case, at least one to three healthy children from the community, with no history of diarrhea in the previous 7 days, were recruited as controls. A single control matched to each LSD case was also recruited. Controls were randomly selected from the neighborhood in which the case resided using the DSS database within 14 days of presentation of the index case. Controls were also matched by age and sex. Written informed consent was sought from the child’s representative before clinical and epidemiological data were obtained and anthropometric measurements were taken. A stool sample was collected from case and control subjects within 12 h of registration for microbiological identification. Additionally, a follow-up home visit was made approximately 60 days after enrollment to assess vital status (Kotloff et al., 2012). Three consecutive attempts were made to re-contact children who were absent at their 60-day visit, in accordance with the study protocol, but if this was unsuccessful, they were considered lost to follow-up. The laboratory procedures undertaken for the extensive microbiological investigations conducted to characterize each diarrheal episode have been described previously (Kotloff et al., 2013, Liu et al., 2016, Panchalingam et al., 2012).

### HIV counseling and testing

Between May 2010 and November 2012, the parents or caretakers of case and control subjects were offered HIV counseling and testing for their children. After providing specific additional informed consent, a blood sample was obtained by finger prick for serological testing on site, according to Mozambican national guidelines. The Determine HIV-1/2 Rapid Test (Abbott Laboratories, Abbott Park, IL, USA) was used. A positive Determine HIV-1/2 result was confirmed using the Uni-Gold Rapid Test (Trinity Biotech Co., Wicklow, Ireland). DNA PCR was required to confirm the HIV infection in children under 18 months of age with a positive HIV serology. HIV infection was diagnosed in a child older than 18 months of age with a positive serological test, or in a child less than 18 months old with a positive DNA PCR. Children testing positive were managed according to the national HIV/AIDS treatment guidelines. For children ≥18 months of age, HIV results became available within approximately 60 min after enrollment in the diarrhea study, whereas for children <18 months of age, the results were only available within the month after enrolment, as a result of delays in PCR confirmation. Children testing positive and confirmed to be HIV-infected were referred for HIV clinical care according to national guidelines.

### Data management and analysis

Overall data were recorded using specific questionnaires, which were checked on site and scanned and sent to a data collection centre in the USA. Queries were resolved on site, and the original source was also stored there. HIV data were collected using a specific form and double-entered into OpenClinica version 2.5 at CISM; discrepancies in the data were resolved by referring to the original form.

Analyses were conducted using Stata/SE software version 13.1 and the package coxphf from R version 3.1.1 (StataCorp, 2013, Ploner and Heinze, 2015, R Core Team, 2014). Appropriate weights based on sampling weights were constructed for the estimation of disease burden, such as the prevalence of HIV in the population and the mortality rate among cases. In parallel, propensity scores estimated by logistic regression were used to adjust these weights for potential non-response bias in HIV status (Rosenbaum and Rubin, 1983, Little and Vartivarian, 1986, Little and Vartivarian, 2003). A crude model of conditional logistic regression with Firth’s penalized likelihood was estimated to assess the association between HIV and MSD, with HIV status as the independent variable. Firth’s penalized likelihood approach is a method of addressing issues of separability, small sample sizes, and bias of the parameter estimates (Firth, 1993, Heinze and Schemper, 2002).

Conditional logistic regression models with Firth’s penalized likelihood were estimated to assess the impact of HIV on the association of each pathogen with MSD or LSD. The modification of the pathogen-specific association with MSD or LSD was assessed by the odds ratio (OR) corresponding to the interaction coefficient, e.g. the ratio and 95% confidence intervals (95% CI) of the OR among HIV-infected children over the OR among the uninfected.

Differences between HIV-infected and HIV-uninfected children in clinical presentations and microbiological etiologies at recruitment were evaluated within the MSD and LSD cases, and statistical tests for independent groups were performed (e.g., Chi-square or Fisher’s exact test for categorical variables and *t*-test for continuous variables).

Mortality analyses were performed within the MSD and LSD cases. A study death was defined as any death occurring in a case or a control within the 60 days up to the follow-up home visit. The time to death (or censoring) was calculated as the difference between the date of death (or 60-day visit), minus the enrolment date, plus the number of days with diarrhea on the day of enrolment.

Kaplan–Meier survival curves were estimated, and the difference between the HIV-infected and HIV-uninfected children was assessed by weighted Cox regression estimation. Mortality rates and appropriate centiles of survival time were also described. Cox regression with Firth’s penalized likelihood was used to estimate the associations with time-to-death adjusted for age. Similarly, the modification of the pathogen-specific association with time-to-death (interaction) was assessed by the hazard ratio (HR) corresponding to the interaction coefficient, e.g. the ratio and 95% CI of the HR among HIV-infected children over the HR among the HIV-uninfected.

### Ethical approval

This study was a site-specific sub-analysis conducted under the framework of the Global Enteric Multicenter Study, a large multicenter study conducted in six other developing countries investigating the burden, etiology, and sequelae of diarrheal disease in infants and young children. The overall protocol and informed consent, including for the HIV sub-study, were both approved by the National Bioethics Committee for Health of Mozambique (CNBS −IRB00002657), the Ethics Committee of Hospital Clínic of the University of Barcelona, and the Institutional Review Board at the University of Maryland. Parents/caregivers of participants provided written informed consent, countersigned by an impartial witness in the case of illiterate parents/caretakers.

## Results

### HIV testing and counseling

**Figure 1:**
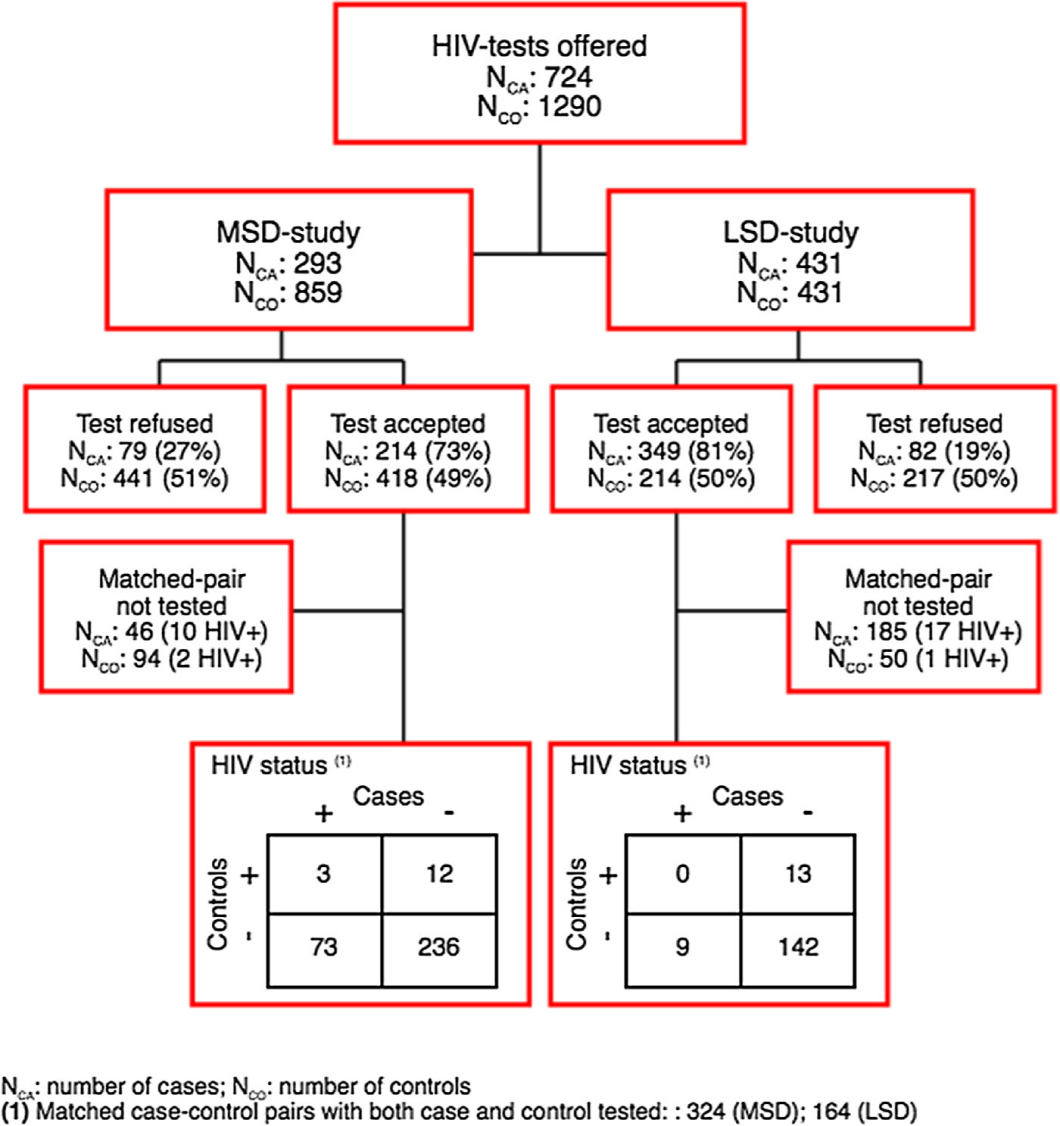
Study profile: HIV counseling and testing among moderate-to-severe diarrhea (MSD) and less-severe diarrhea (LSD) cases and their matched controls.

A total of 2014 children were enrolled during the study period, including 293 MSD cases with their matched 859 controls, and 431 LSD cases with their matched 431 controls. Seventy three percent (214/293) of MSD cases, 49% (418/859) of MSD controls, 81% (349/431) of LSD cases, and 50% (214/431) of LSD controls provided consent for HIV testing, including 324 matched case–control pairs for MSD and 164 matched case–control pairs for LSD (Figure 1).

Overall, HIV prevalence was higher among MSD cases (23%, 49/214; 95% CI 17.72–29.06) than among MSD matched controls (4%, 17/418; 95% CI 2.54–6.46). HIV weighted prevalence increased to 25% in the MSD group (95% CI 17.98–32.60) and decreased to 3% in the control group (95% CI 1.48–7.97). In the 214 MSD cases tested for HIV, males (accounting for 60% of the participants enrolled, 128/214) showed a non-significantly lower HIV positivity rate (26/128, 20%) than females (23/86, 27%) (*p* = 0.2723).

HIV prevalence in the LSD group was similar among cases and controls: 7% (26/349; 95% CI 5.11–10.74) in LSD cases and 7% (14/214; 95% CI 3.90–10.78) in their matched LSD controls. Furthermore, the weighted prevalence rates adjusted for non-response were also very similar: 6% (95% CI 4.28–9.47) for LSD cases and 6% (95% CI 3.00–13.25) for their matched LSD controls.

HIV-infected children had a higher risk of presenting with MSD compared to uninfected children (OR 5.7, 95% CI 3.05–11.32; *p <* 0.0001). This association was not observed in the LSD group, where the HIV infection did not appear to increase the odds of suffering an episode of LSD compared to their matched controls (OR 0.7, 95% CI 0.30–1.60; *p* = 0.403).

### Pathogen profile

After adjusting for HIV, *Shigella spp* (OR 96.8; *p <* 0.0001), rotavirus (OR 4.3; *p <* 0.0001), and Cryptosporidium (OR 2.7; *p* = 0.0040) were all significant risk factors for MSD episodes, whereas Giardia (OR 0.59; *p* = 0.060) and *Clostridium difficile* (positivity for glutamate dehydrogenase antigen (GDH Ag)) (OR 0.24; *p* = 0.0029) were pathogens protective for MSD episodes. Regarding LSD cases, enterotoxigenic *Escherichia coli* producing heat-stable toxin (ETEC of any sequence type (ST); OR 3.2; *p* = 0.0050), enteroaggregative *Escherichia coli* (EAEC, aatA only; OR 10.1; *p* = 0.0373), *Shigella spp* (OR 14.4; *p* = 0.0080), and rotavirus (OR 2.8; *p* = 0.0018) were all risk factors for LSD, whereas Giardia (OR 0.36; *p* = 0.0007), EAEC (aaiC only; OR 0.49; *p* = 0.046), and *C. difficile* (GDH Ag-positive) (OR 0.27; *p* = 0.0023) were the pathogens found to protect against LSD.

For all study cases in which a pathogen was detected, differences according to HIV status were also assessed for every given pathogen. For MSD cases, only Cryptosporidium seemed to border statistical significance, being more prevalent among HIV-positive cases than among HIV-negative ones (31% vs. 18%; *p* = 0.0608), while for LSD cases, only ETEC (any ST) was more frequent among HIV-infected cases (38% vs. 13%; *p* = 0.0016), although Cryptosporidium was also borderline significantly associated with HIV infection (27% vs. 13%, *p* = 0.0759).

An assessment of whether HIV-positive status modified the relationship between pathogens and being a case of MSD or LSD was additionally performed. For MSD, no pathogen significantly increased the risk of being an MSD case among HIV-infected children.

### Clinical characteristics

The analysis of MSD cases showed that HIV-infected children were more likely to be malnourished (defined according to *Z*-scores; 59% vs. 35%; *p* = 0.0020), wasted (33% vs. 19%; *p* = 0.0447), underweight (49% vs. 21%; *p* = 0.0001), or stunted (46% vs. 23%; *p* = 0.0020). Furthermore, they were more likely to be dehydrated with sunken eyes (57% vs. 39%%; *p* = 0.0227) or wrinkled skin (43% vs. 27%; *p* = 0.0381) and more likely to be lethargic or show decreased consciousness (59% vs. 35%; *p* = 0.0026) or fast breathing (37% vs. 21%; *p* = 0.0208). In addition, HIV-infected children were more likely to be admitted to hospital than those not infected (80% vs. 64%; *p* = 0.0435) (Table 1). Of those who were admitted, the mean hospital stay was significantly longer for the HIV-infected: 7.0 days (range 1–24 days) for HIV-infected children vs. 4.3 days (range 0–17 days) for those who were not infected (*p* = 0.0001).

**Table 1:** Clinical characteristics of children less than 5 years of age with moderate-to-severe diarrhea (MSD) at enrollment, according to HIV status.

Variable	HIV-status		*p*-Value
	HIV-uninfected (*n* = 165)	HIV-infected (*n* = 49)	
Weight-for-age *Z*-score	−1.19 (1.35)	−2.14 (1.80)	0.0001
Length/height-for-age *Z*-score	−1.21 (1.23)	−2.08 (1.44)	0.0001
Underweight	34/165 (21%)	24/49 (49%)	0.0001
Malnutrition (*Z*-scores)	57/165 (35%)	29/49 (59%)	0.0020
Sunken eyes	64/165 (39%)	28/49 (57%)	0.0227
Loss of skin turgor	45/165 (27%)	21/49 (43%)	0.0381
Intravenous rehydration	68/165 (41%)	25/49 (51%)	0.2239
Dysentery	34/165 (21%)	3/49 (6%)	0.0186
Hospitalized	106/165 (64%)	39/49 (80%)	0.0435
Vomiting	69/165 (42%)	27/49 (55%)	0.1007
Fever	67/165 (41%)	18/49 (37%)	0.6268
Lethargy or loss of consciousness	58/165 (35%)	29/49 (59%)	0.0026
Convulsions	10/165 (6%)	4/49 (8%)	0.5303
Irritable or restless	21/165 (13%)	5/49 (10%)	0.6669
Belly pain	38/165 (23%)	13/49 (27%)	0.6136
Dry mouth	50/165 (30%)	22/49 (45%)	0.0576
Fast breathing	34/165 (21%)	18/49 (37%)	0.0208

On discharge, a diagnosis of dysentery was less frequent among HIV-infected children with MSD than among uninfected ones (4% vs. 20%; *p* = 0.0082). A diagnosis of malnutrition at discharge was more frequent among HIV-infected children with MSD than among HIV-uninfected cases (16% vs. 6%; *p* = 0.0365), and a diagnosis of invasive bacterial infection was borderline more frequent in the HIV-infected children (4% vs. 0%; *p* = 0.0516) (Table 2).

**Table 2:** Diagnosis at the time of discharge for moderate-to-severe diarrhea (MSD) cases admitted to the hospital, according to HIV status.

Variable	HIV-status		*p*-Value
	Negative (*n* = 165)	Positive (*n* = 49)	
Diarrhea	123/165 (75%)	43/49 (88%)	0.0516
Dysentery	33/165 (20%)	2/49 (4%)	0.0082
Pneumonia/lower respiratory tract infection	28/165 (17%)	9/49 (18%)	0.8203
Malaria	22/165 (13%)	7/49 (14%)	0.8642
Malnutrition	10/165 (6%)	8/49 (16%)	0.0365
Other diagnosis	42/165 (25%)	19/49 (39%)	0.0697
Multiple diagnoses	77/165 (47%)	30/49 (61%)	0.0735

Table 3 summarizes the clinical characteristics of LSD cases at enrollment, according to HIV status. HIV-infected children were more likely to be malnourished (62% vs. 28%, *p* = 0.003) or stunted (58% vs. 23%; *p* = 0.0001) than uninfected ones. The stool characteristics of the HIV-infected children were different to those of the HIV-uninfected (*p* = 0.0187): they less frequently presented with simple watery diarrhea (58% vs. 78%) and more commonly showed pathological products in their stool (mucus, 42% vs. 22%).

**Table 3:** Clinical characteristics of children less than 5 years of age with less severe diarrhea (LSD) at enrollment, according to HIV status.

Variable	HIV		*p*-Value
	Negative (*n* = 323)	Positive (*n* = 26)	
Weight-for-age *Z*-score	−0.73 (1.21) [323]	−1.12 (1.33) [25]	0.1282
Length/height-for-age *Z*-score	−1.17 (1.19) [323]	−1.63 (1.51) [26]	0.0688
Underweight	44/323 (14%)	7/25 (28%)	0.0719
Stunted	74/323 (23%)	15/26 (58%)	0.0001
Malnutrition (*Z*-scores)	89/323 (28%)	16/26 (62%)	0.0003
Vomiting	89/323 (28%)	10/26 (38%)	0.2352
Fever	91/323 (28%)	9/26 (35%)	0.4846
Lethargy	80/323 (25%)	6/26 (23%)	0.8474
Lethargy or loss of consciousness	72/323 (22%)	5/26 (19%)	0.7173
Belly pain	61/323 (19%)	3/26 (12%)	0.4397
Dry mouth	3/323 (1%)	1/26 (4%)	0.2673
Fast breathing	24/323 (7%)	1/26 (4%)	1.0000
Stool characteristics at enrollment			
Simple watery	252/323 (78%)	15/26 (58%)	0.0187
Sticky/mucoid	71/323 (22%)	11/26 (42%)	–

### Mortality analysis

**Figure 2:**
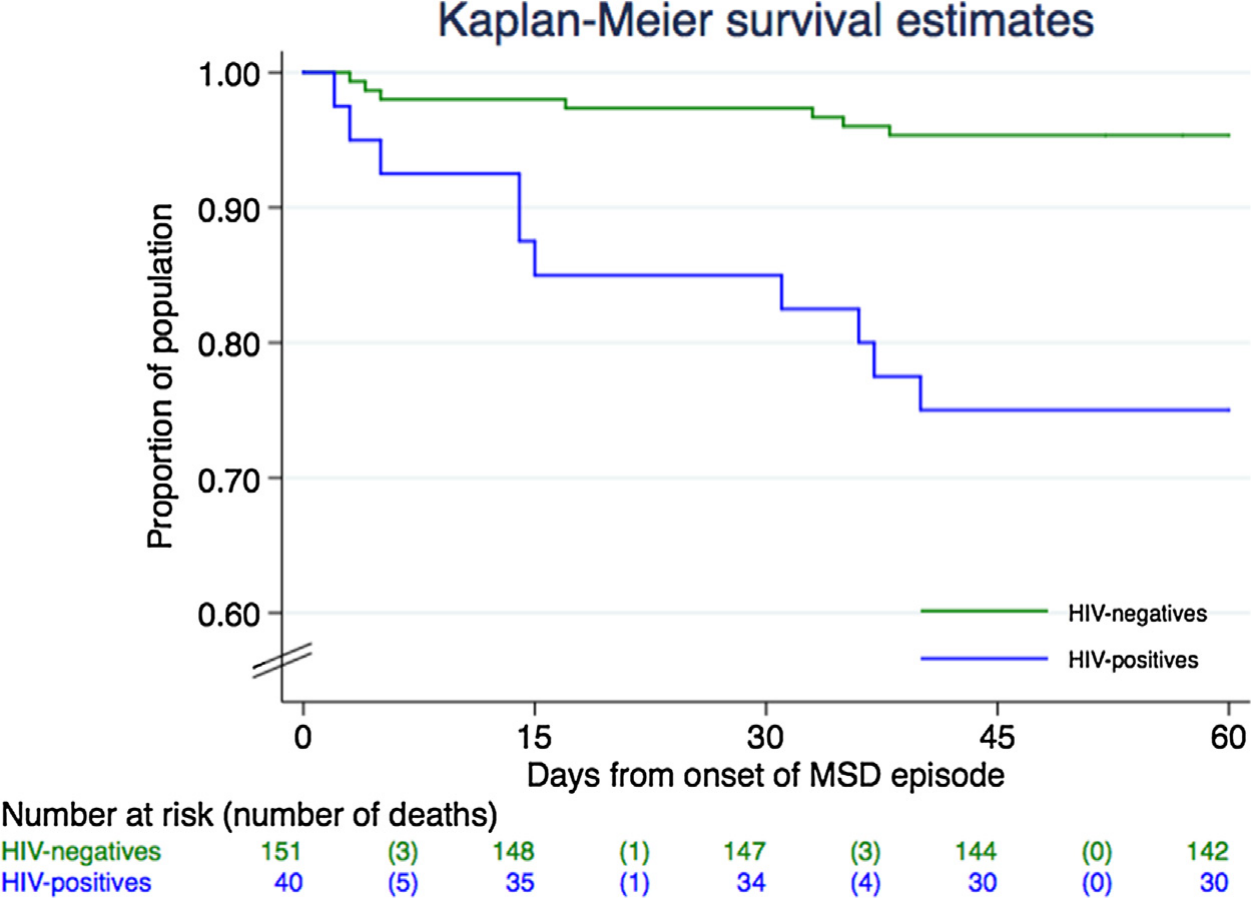
Survival at the 60-day follow-up home visit according to HIV status among moderate-to-severe diarrhea (MSD) cases.

Of all MSD cases with a known HIV status, 89% (191/214) successfully completed their 60-day follow-up home visit. Seventeen deaths occurred among the study participants: 25% (10/40) among the HIV-infected children and 5% (7/151) among the uninfected. Three deaths occurred in the LSD group: 4% (1/25) of the HIV-infected children died, compared to 0.6% (2/303) of the HIV-uninfected. Figure 2 shows the Kaplan–Meier survival curve for children with MSD according to HIV status.

Mortality rates were higher among HIV-infected cases with MSD than among the uninfected: 33.84 (95% CI 15.28–81.04) vs. 5.49 (95% CI 1.92–21.41) deaths/1000 person-weeks at risk (*p* = 0.0039). The difference in infant mortality rates was not statistically significant (11.50, 95% CI 3.73–50.20 vs. 40.33, 95% CI 15.03–135.82 deaths/1000 person-weeks at risk; *p* = 0.08), while in the other age groups a larger difference was observed between HIV-infected and HIV-uninfected children, although it could not be evaluated due to the low (or null) number of deaths among the HIV-uninfected children.

According to the survival time centiles, 5% of the HIV-infected children had already died at 3 days after the onset of the MSD episode. This was not observed in the HIV-uninfected until 38 days after the onset of the episode. Similarly, mortality rates were higher among HIV-infected children with LSD than among the uninfected (5.09 vs. 0.58 deaths/1000 person-weeks at risk, respectively), although the difference did not reach statistical significance (*p* = 0.0748).

Finally, it was aimed to investigate the specific effect of the different pathogens (*n* = 29) analyzed as part of the study in terms of the likelihood of affecting survival among MSD and LSD cases. None of the pathogens seemed to have an independent effect in terms of modifying the risk of death, neither for MSD nor for LSD cases.

## Discussion

This article describes a novel approach that was used to explore the role of HIV in diarrheal disease, based on a very robust case–control study design, which adds an important level of confidence to the results. The findings confirm that HIV infection plays a significant role in the epidemiology of MSD and its associated mortality in this particular rural area of southern Mozambique, therefore explaining the high mortality rates reported previously (Kotloff et al., 2013, Sow et al., 2016). The strong association between HIV infection, MSD, and mortality in children is in accordance with the findings of previous studies (Irena et al., 2011).

The overall high prevalence of HIV infection (25%) among children with MSD seeking care at health centers is in line with data from other studies recruiting sick children within the health system (34% of the children in a pneumonia study being HIV-positive (Bassat et al., 2011)), and with the prevalence rate observed among pregnant women during antenatal consultations in Manhiça District (29%) (Gonzalez et al., 2012), in spite of the many advances in the implementation of mother-to-child vertical transmission programs conducted in the whole country, including in Manhiça District. It therefore appears crucial to continue pushing for the improvement and scale-up of such programs (including monitoring adherence, as up to 25% of children born to HIV-positive mothers are infected in the first year of life), along with other strategies designed to decrease the consequences of HIV infection, such as the scale-up of ART among infected children. This would likely reduce not only the frequency of diarrheal episodes but also the complications that are often observed among HIV-infected children. These findings also corroborate the results of previous studies, in which HIV infection was shown to increase the risk of diarrhea, worsening the symptoms and prolonging its duration (Merchant and Lala, 2012).

One of the relevant findings of this study was that the likelihood of developing an MSD episode was strongly associated with being HIV-positive (OR 5.68; *p <* 0.0001), while the likelihood of developing an LSD episode was not. In this respect, HIV infection may condition the host’s response to pathogens causing diarrhea, commonly triggering more severe episodes. The fact that differences in relation to HIV as the cause of the more ordinary LSD episodes were not identified may also reflect diarrheal infections affecting those hosts with more recently acquired HIV infections, and therefore less immunosuppression, or otherwise occurring among slow progressors for AIDS. In any case, the World Health Organization now recommends systematic HIV counseling and testing as part of the Integrated Management of Childhood Illness (IMCI) (World Health Organization, 2013), for all children seeking care in health centers, irrespective of the motive of consultation, as a better approach to readily recognizing HIV-infected children and thus triggering appropriate management, and thereby improving the prognosis and reducing the incidence of opportunistic diseases, complications, and deaths.

In this study, Cryptosporidium was among the non-bacterial pathogens most frequently detected, and this organism was associated with an increased risk of MSD among HIV-infected children. This finding is in concordance with the results reported in previous studies (Adamu et al., 2013, Mathur et al., 2013, Paboriboune et al., 2014, Ramakrishnan et al., 2007, Tumwine et al., 2005). The study was conducted in an area where malnutrition is highly endemic (Instituto Nacional de estatistica (INE), 2008, Nhampossa et al., 2013), and such a pre-existing nutritional impairment, even in the absence of HIV infection, may have negatively affected the immune systems of the children, possibly leading to an increase in the incidence of Cryptosporidium infections, thereby explaining the diagnosis of these parasites even among HIV-negative children. However, Cryptosporidium and other parasites have more traditionally been described among chronic and persistent diarrhea cases in HIV-infected patients (Mathur et al., 2013, Paboriboune et al., 2014). The recruitment of subjects into this study was restricted to acute cases – no stool samples were collected from persistent or chronic diarrhea cases.

This study also found Shigella and rotavirus to be pathogens with a strong and significant association with MSD according to HIV status. Other studies conducted in Africa have reported similar evidence, with the incidence of rotavirus being higher among HIV-infected children than among those not infected (Groome and Madhi, 2012b). Another study also conducted in the same African region, documented a relatively higher prevalence of Shigella among HIV-infected individuals when compared to uninfected ones (Rowe et al., 2010). In the present study, the number of Shigella isolated was very small, probably explaining the scarcity of negative outcome events. Conventional logistic regression is not a good option for this type of data, and for this reason it was analyzed using penalized estimation. Despite this, the ORs remained very large (OR 96.8) and fairly implausible. However, it is believed that they are interesting results that suggest a very strong association, as also reported in other studies.

The associations of pathogens and LSD, in the context of HIV status, were also similar to those of MSD, although Cryptosporidium was not found to be a significant risk factor, while ETEC (any ST) and EAEC (aatA only) were positively associated.

Certain pathogens in this case–control study were found to be negatively associated with the risk of MSD or LSD, even after adjusting for HIV. Indeed, *Giardia lamblia* and *C. difficile* infection (determined by positivity for GDH antigen) were significantly associated with protection against MSD and LSD, and EAEC (aaiC only) was found to be protective against LSD episodes only. For Giardia, these results are in concordance with the general findings of the GEMS (Kotloff et al., 2012) and other similarly designed studies (Tellevik et al., 2015), whereas Giardia was identified significantly more frequently in controls than in patients with MSD. Although some authors have suggested that this pathogen may be a significant risk factor for diarrhea among HIV-infected individuals (Moolasart, 1999), this remains to be substantiated. Pavlinac et al. described Giardia infections as occurring more frequently among HIV-uninfected individuals than in infected individuals (Pavlinac et al., 2014).

Regarding *C. difficile* infection, the results are more difficult to interpret. The prolonged use of antibiotics has been described as a clear risk factor for this infection (Bignardi, 1998). *C. difficile* is a bacterium that is naturally present in the intestinal flora of about 3% of adults and 66% of children. Such bacteria do not generally cause problems among healthy people; however, some antibiotics used to treat other health conditions may significantly alter the balance of ‘good bacteria’ in the intestinal flora. When this happens, *C. difficile* can multiply and cause symptoms such as diarrhea. In contrast to what would be expected in Manhiça District, an area of high HIV endemicity where antibiotics such as co-trimoxazole are used in a massive and prolonged way among HIV-infected individuals for the prevention of opportunistic infections, *C. difficile* infections were negatively associated with the incidence of MSD episodes when adjusted by HIV status. A possible explanation for this is that in such a population, the use of antibiotics favors the proliferation of *C. difficile* infections among healthy individuals (controls) rather than the proliferation of *C. difficile*-associated disease (cases). Other studies have reported a high prevalence of *C. difficile* infections and associated disease among HIV-infected individuals (Haines et al., 2013), or an increased incidence of *C. difficile* in the general population (Rupnik et al., 2009). Further research will be needed to clarify the role of *C. difficile* infections as a risk or protective factor for MSD among HIV-infected individuals.

The study also revealed an interesting finding in relation to the decreased association of dysentery (both on admission but also upon discharge from hospital) with HIV infection, in the context of MSD. This may also be related to the high intake of prophylactic antibiotics among HIV-infected individuals, which may have contributed to the reduction in dysentery episodes.

### Study limitations

Several limitations need to be taken into consideration when interpreting the results of this study. While interpreting the effect of HIV on diarrheal diseases, it would have been useful to have more detailed information on the immunosuppression status of all confirmed HIV cases. Unfortunately, the study did not collect data on (1) the use and duration of ART, (2) the use of co-trimoxazole as prophylaxis for opportunistic infections, or (3) CD4 counts or HIV viral load, which could have allowed a more detailed evaluation of the degree of immunosuppression and how this or any given treatment could have influenced the incidence and prevalence of enteric pathogens in HIV-infected children. Moreover, the fact that HIV testing was not mandatory and required an additional patient consent, led to a considerable proportion of participants not having HIV results − MSD and LSD cases and their respective controls − hindering the interpretation of the study results.

## Conclusions

The prevalence of HIV remains high among children with MSD in Manhiça District despite the efforts made in the implementation of programs for the prevention of vertical transmission and the roll-out of ART for those children diagnosed to be HIV-infected. HIV infection was found to increase the risk of having MSD caused by rotavirus, Shigella, and Cryptosporidium.

These findings emphasize the need for routine screening for HIV infection among children presenting with diarrhea, as the prognosis is worse and the mortality risk is higher in HIV-infected children with this symptomatology. These findings may additionally support the need to strengthen all efforts to prevent vertical transmission in the antenatal clinics, a critical step to reduce HIV transmission and thus all the undesirable consequences.
